# Assessing Trauma Management in Urban and Rural Populations in Norway: A National Register-Based Research Protocol

**DOI:** 10.2196/30656

**Published:** 2022-06-17

**Authors:** Inger Marie Waal Nilsbakken, Stephen Sollid, Torben Wisborg, Elisabeth Jeppesen

**Affiliations:** 1 Department of Research Norwegian Air Ambulance Foundation Oslo Norway; 2 Faculty of Health Sciences University of Stavanger Stavanger Norway; 3 Division of Emergencies and Critical Care Norwegian National Advisory Unit on Trauma Oslo University Hospital Oslo Norway; 4 Department of Anaesthesiology and Intensive Care Hammerfest Hospital Hammerfest Norway; 5 Interprofessional Rural Research Team – Finnmark Faculty of Health Sciences University of Tromsø – Arctic University of Norway Tromsø Norway

**Keywords:** trauma, emergency medicine, prehospital care, trauma registries, epidemiology, quality of health care

## Abstract

**Background:**

Time is considered an essential determinant in the initial care of trauma patients. In Norway, response time (ie, time from dispatch center call to ambulance arrival at scene) is a controversial national quality indicator. However, no national requirements for response times have been established. There is an ongoing debate regarding the optimal configuration of the Norwegian trauma system. The recent centralization of trauma services and closure of emergency hospitals have increased prehospital transport distances, predominantly for rural trauma patients. However, the impact of trauma system configuration on early trauma management in urban and rural areas is inadequately described.

**Objective:**

The project will assess injured patients’ initial pathways through the trauma system and explore differences between central and rural areas in a Norwegian trauma cohort. This field is unexplored at the national level, and existing evidence for an optimal organization of trauma care is still inconclusive regarding the impact of prehospital time.

**Methods:**

Three quantitative registry-based retrospective cohort studies are planned. The studies are based on data from the Norwegian Trauma Registry (NTR; studies 1, 2, and 3) and the local Emergency Medical Communications Center (study 2). All injured patients admitted to a Norwegian hospital and registered in the NTR in the period between January 1, 2015, and December 31, 2020, will be included in the analysis. Trauma registry data will be analyzed using descriptive and relevant statistical methods to compare prehospital time in rural and central areas, including regression analyses and adjusting for confounders.

**Results:**

The project received funding in fall 2020 and was approved by the Oslo University Hospital data protection officer, case number 18/02592. Registry data including approximately 40,000 trauma patients will be extracted during the first quarter of 2022, and analysis will begin immediately thereafter. Results are expected to be ready for publication from the third quarter of 2022.

**Conclusions:**

Findings from the study will contribute to new knowledge regarding existing quality indicators and with an increasing centralization of hospitals and residents, the study will contribute to further development of the Norwegian trauma system. A high generalizability to other trauma systems is expected, given the similarities between demographical changes and trauma systems in many high-income countries.

**International Registered Report Identifier (IRRID):**

PRR1-10.2196/30656

## Introduction

### Background

Traumatic injuries constitute a major global health problem [1]. According to the Global Burden of Disease study conducted by the World Health Organization in 2013, 973 million people sustained injuries that required health care, and injuries accounted for 4.8 million deaths. Although mortality from injuries has been reduced in the last four to five decades because of injury prevention and better trauma care, it is still one of the leading causes of mortality and morbidity among younger age groups [2].

There is broad agreement that a well-functioning trauma system, with a seamless treatment chain from accident site to completed rehabilitation, is essential for optimal patient outcomes [3]. In November 2015, the Norwegian government published a new health and hospital plan that aimed at ensuring a coherent system of emergency services in and outside hospitals that provides adequate security and quality of health care throughout the country [4]. Parallel to this, an updated national trauma plan has been developed and implemented. The plan includes all stages of the chain of survival, from first aid at the scene of injury; criteria for suspecting serious injury; and destination for definite care, treatment, and rehabilitation [5,6].

In Norway, the scattered population, long distances, seasonal cold, and a rough climate challenge the organization and provision of acute care medical services [7]. There is an ongoing debate regarding the optimal configuration of today's trauma systems, and there has been a tendency toward the centralization and closures of emergency hospitals with trauma wards. In 2002, 52 hospitals had a trauma ward, and today, there are only 38 hospitals with one. The debate must be held considering the increased availability of advanced prehospital treatment, which might counteract the long transport time for trauma patients. More centralized emergency medical competence may affect where the patient is transported and treated.

### Prehospital Time

As the distance between injury site and trauma center increases, the choice between whether to transport patients directly to definite care or stabilize patients either at the accident scene or in nontrauma center hospitals with a subsequent transfer to a trauma center becomes increasingly pertinent [8].

Several studies on the effect of prehospital transport time on mortality have been conducted in the last two decades. In 2020, a scoping review by Bedard et al [9] on the effect of prehospital time on trauma outcomes was published. They reported on positive, negative, and neutral associations between prehospital time and inhospital mortality. The relationship between prehospital time and mortality thus seems to be unclear. However, most of the included studies in this scoping review did not differentiate between blunt and penetrating trauma. Other studies, including a systematic review by Harmsen et al [10] in 2015 and individual empirical studies, have found a clear positive effect of prehospital time on survival for penetrating and traumatic brain injuries [11]. In the case of blunt injuries, the results remain mixed. In the same systematic review, short emergency response time and transport time from scene to hospital were associated with better survival. Moreover, a longer on-scene time had favorable odds for survival [10]. On the contrary, Waalwijk et al [12] found an association between prolonged on-scene time and mortality in their recently published article.

Furthermore, studies included in the 2020 scoping review by Bedard et al [9] were largely based on urban areas with a high population and hospital density. For rural areas, both the incidence and the consequence of traumatic injury exceed those of urban areas, while evidence for the optimal organization of trauma care is less conclusive. By comparison, a large 4-year registry study from a trauma register in Quebec, Canada, reported on mortality differences between rural and urban areas. They collected data from nearly 80,000 registered trauma patients and concluded there was an increased mortality in rural areas [13]. These findings suggest that rural areas are associated with higher mortality due to longer prehospital times.

### Norwegian Trauma System

Norway has a scattered population of 5.4 million people [14]. Approximately 80% of its inhabitants live in urban areas, while the rest live in rural areas [15]. It is a high-income country with a publicly funded health care system and a national trauma system.

According to the national trauma plan, 34 acute care trauma hospitals and 4 trauma centers receive and treat trauma patients in Norway. All acute care trauma hospitals offer general surgical and orthopedic services and are capable of stabilizing severely injured patients before transferring them to trauma centers if necessary. The acute care trauma hospitals do not offer neurosurgery, intervention radiology (except for a few), or other specialized services. The trauma centers offer all medical specialties, including neurosurgery, and can manage all types of injuries. Both emergency hospitals and trauma centers have criteria for trauma team activation (TTA). In addition, there are several competence requirements for trauma team members, including passing an Advanced Trauma Life Support course and having a minimum of 4 years of surgical experience for the team leader [6,16].

In Norway, emergency medical communications centers (EMCCs) are organized as several public centers spread across the country with their own emergency contact number. The emergency call receivers use predefined criteria for triage and dispatch of resources based on the caller’s information.

#### National Quality Indicators

The Norwegian trauma plan defines the following quality indicators related to patient transport:

- Rate of patients with transport time to trauma center less than 45 minutes (otherwise the patient should go to an emergency hospital with a trauma ward)
- Proportion of correct destination from scene for all patients with a suspected serious injury

Norwegian authorities have defined national quality indicator for prehospital *response time* as the time interval from when an EMCC is notified until the ambulance arrives on scene [17]. Until recently, this quality indicator was merely a recommendation, but in March 2021, the Norwegian Parliament agreed on a resolution to fix the response time by law [18]. The quality indicator for response time is as follows:

- In urban areas, the ambulance should arrive at the scene within 12 minutes in 90% of emergency events.
- In rural areas, the ambulance should arrive at the scene within 25 minutes in 90% of emergency events.

### Aim

The overall aim of the project is to assess how trauma system configurations in urban and rural areas affect the initial management of trauma patients. Injured patients’ initial pathways through the Norwegian trauma system will be described, and urban-rural differences will be explored. We will also determine the association between prehospital time and outcomes in trauma patients.

First, the project will examine dispatch time, prehospital time, interventions given, patient destination, and modes of transport in a Norwegian trauma population with data from the Norwegian Trauma Registry (NTR) for 2015 to 2020. Differences in gender and age will be examined along with injury mechanism. Second, we will investigate to what extent ambulance services, including emergency medical services (EMS) and helicopter EMS (HEMS), reach severely injured patients within an acceptable time frame according to national quality indicators. Third, we will investigate the time spent on primary care in acute care trauma hospitals compared to trauma centers and time spent transferring patients between hospitals and trauma centers. For one of our studies, we will investigate response dispatch for severely injured patients (Injury Severity Score [ISS]>15) with additional data from the EMCC journal [19].

## Methods

This project consists of 3 quantitative registry-based retrospective cohort studies using data from the NTR studies 1, 2, and 3 and the local EMCC data (study 2).

### Hypotheses and Objectives

The specific objectives and hypotheses of the project are as follows.

#### Study 1

The following are the objectives, outcome measures, and hypothesis for study 1:

- Objectives
  To assess how response times to suspected severely injured patients comply with national quality indicators [17]
    Examine differences between urban areas compared to rural areas using the Statistics Norway centralization index [20]
    Compare prehospital time for: (1) primary admissions to acute care trauma hospitals and (2) primary admission to trauma centers
    Examine transfers between hospitals
- Primary outcome measures
  Response time: time interval from dispatch to ambulance at scene
- Secondary outcome measures
  Time spent at scene
  Prehospital time for primary admissions to emergency departments (ED) at trauma centers
  Prehospital time for primary admissions to ED at acute care trauma hospitals.
  Total prehospital time from dispatch to ED admission at trauma centers and acute care trauma hospitals
  Time from primary to secondary hospital admission
- Hypothesis: We hypothesize shorter response time in urban areas compared to rural areas.

#### Study 2

The following are the objectives, outcome measures, and hypothesis for study 2:

- Aim
  To conduct an in-depth analysis of dispatch dynamics in a subgroup of severely injured patients (ISS≥9/ISS> 15) and explore potential differences in urban and rural areas by linking data from the NTR with local EMCC data. The decision-making process at the EMCC is an unexplored but important part of the chain. The analysis will include:
    Coherence between initial information and patient physiology (including Glasgow Coma Scale)
    Evolution of initial and subsequent resource utilization
    Prehospital medical interventions according to level of service provider
- Primary outcome measures
  Response
  Emergency
  Resources utilized
  Triage (triage to hospital and triage to TTA)
- Secondary outcome measures
  More precise location data
- We will look at the possibility of cooperation with other Nordic counties.
- Hypothesis: Over- and undertriage occur to a greater extent in patients with moderate to severe injuries. In the trauma system, accuracy is difficult both in relation to where the patient is to be transported and in connection with TTA.

#### Study 3

The following are the objectives, outcome measures, and hypothesis for study 3:

- Aim
  To explore potential differences in quality of trauma care in severely injured patients between urban and rural areas.
  To examine ground EMS compared to HEMS on scene competence, interventions given, and transport type for patients with severe injury (ISS≥9/ISS>15).
- Primary outcome measures
  Mortality
  Length of hospital stay/intensive care day/intubations days
  The Glasgow Outcome Scale Extended/The American Society of Anesthesiologists physical status classification [21].
- We will adjust for the following possible confounding factors:
  Gender/age
  Injury severity
  If the data quality allows it, it may be relevant to adjust for physiological data.

We may consider extracting supplementary data from the Norwegian Cause of Death registry to verify the mortality rate.

Due to the exploratory design of the study, no hypothesis is formed.

### Study Setting

This a national population-based study including the entire mainland of Norway and its population. The emphasis is initial trauma management including the prehospital phase and emergency departments belonging to the 34 acute care trauma hospitals and 4 trauma centers that comprise the Norwegian trauma system.

### Study Population

All injured patients admitted to a Norwegian hospital and registered in the NTR between January 1, 2015, and December 31, 2020, will be included in the analysis. Textbox 1 shows the inclusion and exclusion criteria. The registry data will include approximately 40,000 trauma patients.

**Inclusion and exclusion criteria.**

**Inclusion criteria**

- All patients admitted with trauma team activation (TTA) on arrival to the emergency department in all acute care trauma hospitals and trauma centers in Norway, irrespective of Injury Severity Score (ISS) and New Injury Severity Score (NISS)
- All patients treated at an acute care trauma hospital or trauma center in Norway without TTA and with one or more of the following injuries:
  Penetrating injury to the head, neck, torso, or extremities proximal to elbow or knee
  Head injury with Abbreviated Injury Score ≥3
  NISS>12
- All patients with trauma-related death at site of trauma or during transportation to hospital who are referred to hospital, but where prehospital management/treatment was initiated

**Exclusion criteria**

- Patients with chronic subdural hematoma without other trauma-related injuries
- Patients with injuries from drowning, inhalation, hypothermia, and asphyxia without concomitant trauma
- Patients who die on scene without the activation of prehospital resources.
- “Walk-in” traumas, meaning patients who present to hospital via private vehicle, police vehicle, or other/unknown
- Patients who are not registered with the emergency medical communications centers

### Variables

#### Time Variables

**Figure 1:**
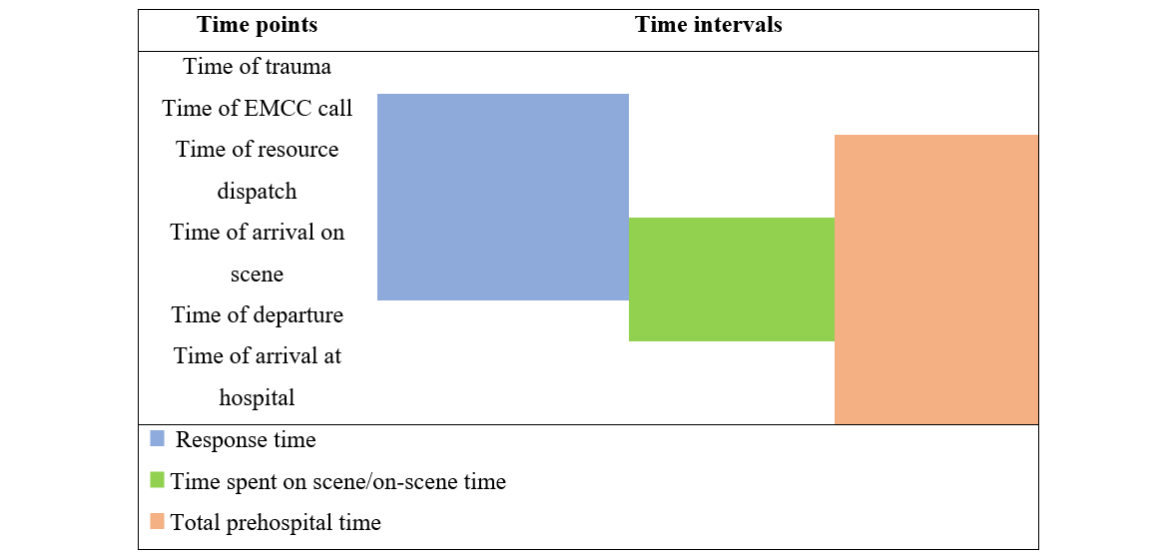
Time points and time intervals illustrated.

The primary variables are time intervals in different prehospital phases determined by time points extracted from the NTR. The time points include time of trauma, time of EMCC call registration, time of resource dispatch, time of arrival on scene, time of departure, and time of arrival at a hospital (Figure 1).

#### Patient Characteristics

Secondary variables describe the study population and include:

- Age, gender, preinjury health status, injury mechanism, ISS, New Injury Severity Score (NISS), discharge health state, discharge destination, and mortality [22]
- Municipal code for further determination of centralization index
- Physiological data (prehospital and emergency room data)
- Prehospital data: prehospital stabilizing interventions, prehospital treatment level, and transport type
- Intrahospital data: emergency department stabilizing interventions

#### Triage

For our one-year cohort study from a selection of hospitals, the variable triage (triage to hospital and triage to TTA) will also be investigated. The aim is to examine the accuracy of resource utilization, triage, and severity of injuries of trauma patients.

### Data Analysis

Registry data for studies 1 and 2 will be analyzed using descriptive statistical methods and relevant statistical methods to compare prehospital time in rural and central areas. The studies will comply with the STROBE (Strengthening the Reporting of Observational Studies in Epidemiology Statement) checklist. Categorical variables will be analyzed with Pearson chi-square test, continuous variables with normal score distribution with *t* tests, and skewed distributions with the Mann-Whitney *U* test. We will consider using Fisher exact test for smaller subgroups. For study 3, we intend to use logistic regression analysis for the dependent variable mortality. Independent variables will be centralization index, gender/age, ISS, and likely physiological data. This model will allow us to identify the effect of rurality/centrality (centralization index) on patient mortality, adjusted for covariates. The strength of association will be reported as an odds ratio (OR) with 95% CIs. Low statistical power due to small groups and few events could result in some significant differences with broad 95% CIs. We plan to test correlations between the centralization index with Spearman rank correlation test. The significance level is set at *P*<.05. The analyses will be performed with SPSS software version 27 or higher (IBM Corp).

### Ethics Approval

Research will be conducted according to the ethical guidelines of the Helsinki Declaration. The study protocol and delivery of data according to the March 2020 application were approved by the Oslo University Hospital data protection officer (number 129324), who is the data controller for the NTR. After assessment, the study was exempted from formal ethical approval by the regional committees for medical and health research because it is health service research and thus is not required to be presented. The NTR has concession from the Norwegian data protection authority to include patients without their consent because large parts of those included have temporarily limited consent competence upon contact with the health trust. Nevertheless, all patients have a reservation right, which means that patients can withdraw consent to be registered. For study 2, we will apply for approval from the regional committee for medical health research to collect local EMCC data.

## Results

According to its annual report for 2019, the NTR registered 7948 patients that same year. Several patients were treated at more than one hospital; therefore, the total number of trauma records is higher at 8788. This is due to the organization of the trauma system in Norway, where patients are often transported to the nearest emergency hospital for initial stabilization before being transferred to a trauma center. According to the same report, approximately 13% (n=1051) of the patients had an ISS>15 and approximately 21% (n=1689) had an NISS>15. Of the patients, a total of 67% were male, and the age group with the highest incidence of trauma was 16-24 years for both genders. The average age was 43 (median 44) years. Motor vehicle accidents were the most common cause of trauma (45%), followed by falls (42%) and sports injuries (22%) [23].

Based on the data from 2019 and the annual reports from the years prior, we assume we will extract data on approximately 40,000 patients registered in the NTR for further analysis, and this will be sufficient for us to carry out statistical analyses and draw our conclusions. Power analysis will be carried out for each individual study and outcome measure. For some subgroup analyses and outcome measures, we will consider doing power analysis before further analyses.

## Discussion

### Summary

We aim to explore to what extent ambulance services (including HEMS) in Norway reach severely injured patients within an acceptable time frame according to national quality indicators. We also want to examine differences between prehospital time, interventions, and triage in urban and rural areas in Norway. This field is unexplored at the national level, and existing evidence for the optimal organization of trauma care is still inconclusive regarding the impact of prehospital time. There is an ongoing debate on the relevance and importance of prehospital time in Norway.

### Relevance

Findings from this study will contribute to new knowledge regarding existing quality indicators, and with the increasing centralization of hospitals, this study will contribute to the further development of the Norwegian trauma system. The project adheres closely to the thematic priorities of the call to generate new knowledge about structural, organizational, and economic factors that impede and promote integrated, coherent patient and user pathways to trauma patients and services. Given the similarities between demographical changes and trauma systems in many high-income countries, we expect that our study findings will have an impact on other trauma systems outside of Norway.

### Strengths and Limitations

The NTR is a national quality registry that provides information about potentially severely injured patients in Norway. The main objective of the registry is to monitor trauma treatment and contribute to an increased quality of trauma care throughout the country [23]. This makes the registry well designed for research. There are several strengths in these studies: data needed for analysis already exist, and the data collection has been done independently of the study. A large sample size gives good statistical power and will help detect small effect sizes and true differences [24].

According to the NTR’s annual report for 2019, all 38 hospitals with a trauma ward in Norway delivered data to the registry, and the coverage is estimated to be >95% [23].

Limitations are inherent to the retrospective design of the quantitative studies, with a risk of bias and the fact that causal factors cannot be explored. As registers may be missing data on important factors, this research design may be prone to confounding errors [24].

The NTR specifically has known deficiencies in prehospital physiologic data due to missing data and, to some extent, coding and import issues. The challenge of collecting prehospital physiological parameters exists for many countries [25]. Severely injured patients in this project will thus be selected based on injury severity (ie, retrospective determination of ISS). These data obviously were not apparent at the scene, leading to suboptimal possibilities for the selection of destination, treatment priorities, and provider level.

Hospital-based registry data are very likely to cause a risk of selection bias. The majority who die following trauma die prehospitally, and the proportion of prehospital deaths is higher in rural than urban areas [26,27]. We can assume that patients are included in data registries as “survivors” to a greater extent in rural areas, as opposed to central areas where truly unstable patients die to a greater extent in hospital.

#### Dissemination Plan

Findings from the studies will be presented at national and international conferences and published in three peer-reviewed international medical journals.

## Abbreviations

ED: emergency department
EMCC: emergency medical communications centers
EMS: emergency medical services
HEMS: helicopter emergency medical services
ISS: Injury Severity Score
NISS: New Injury Severity Score
NTR: Norwegian Trauma Registry
OR: odds ratio
STROBE: Strengthening the Reporting of Observational Studies in Epidemiology Statement
TTA: trauma team activation
